# Neonatal encephalopathy—controversies and evidence

**DOI:** 10.1111/aogs.14416

**Published:** 2022-08-03

**Authors:** Nishita Mehta, Prakesh Shah, Amar Bhide

**Affiliations:** ^1^ Department of Obstetrics and Gynecology St George's University Hospital NHS Foundation Trust London UK; ^2^ Department of Neonatology Mount Sinai Hospital Toronto Canada

Neonatal encephalopathy (NE) is a “clinically defined syndrome of disturbed neurologic function in the earliest days of life in an infant born at or beyond 35 weeks of gestation, manifested by a subnormal level of consciousness or seizures, and often accompanied by difficulty with initiating and maintaining respiration and depression of tone and reflexes” as defined by the Task Force on Neonatal Encephalopathy.^1^ This description has been endorsed by various international bodies. Neonatal encephalopathy and its subset, hypoxic ischemic encephalopathy (HIE) are well‐recognized conditions which have been studied extensively. Sarnat and Sarnat described grades of neonatal encephalopathy in 21 neonates; however, it is noteworthy that all those infants showed evidence of hypoxemia.^2^ Now it is understood that NE has many causes and hypoxemia‐ischemia is one of them. The grading scale, that was intended for non‐progressive encephalopathies in which the injury occurred at or some time before birth, is now complete and not ongoing. In the perinatal‐neonatal field, several efforts are underway to resolve two important questions with some clarity emerging from existing studies; however, a dilemma persists.

## 1 WHAT PROPORTION OF NE CASES ARE ASSOCIATED WITH EVIDENCE OF HYPOXEMIA/ISCHEMIA?

A study from Western Australia, by Badawi et al.^3^ compared 164 term infants with clinical features of moderate to severe encephalopathy to 400 non‐encephalopathic infants identified over 15 months. Data on antepartum and intrapartum risk factors were collected and compared. Due to the retrospective nature of the study, certain variables were available (eg cord prolapse and uterine rupture); however, some other key variables were missing (eg cardiotocograph abnormalities, cord gases). Intrapartum risk factors were adjusted for the presence of antenatal risk factors. The authors reported that most cases (69%) in their cohort had only antepartum risk factors associated with the occurrence of NE. Presence of only intrapartum risk factors were identified in only 5% of cases. This study concluded that antepartum risk factors were associated with the majority of cases of NE.^3^, ^4^ However, there were certain limitations to the study, such as the lack of cord gases to detect intrapartum hypoxia, and the non‐contemporaneous nature of the study.

Nelson et al. in another retrospective cohort study of 4165 singleton neonates of >36+0 weeks, from the Vermont Oxford Network Neonatal Encephalopathy Registry^5^ reported that a preexisting medical condition was present in 27% of cases; however, they included maternal hypertension and diabetes. Similarly, a preexisting fetal compromised state including small‐for‐gestational age was identified in 25% of cases. Overall, pre‐labor maternal or fetal condition associated with fetal compromise was identified in 1906/4165 (46%) of cases. In this cohort nearly half of the cases had data on cord gases available; over half of these cases had a cord pH <7.09, and nearly half had a cord blood base deficit >12. From this study it appears that about half of all cases of encephalopathy suggest the presence of hypoxemia at birth. This proportion is significantly higher than that reported by Badawi et al. It is possible that in the study by Badawi et al., adjustment for antepartum factors leads to intrapartum factors losing their significance if the two are highly correlated.

Cowan et al. studied brain magnetic resonance imaging (MRI) or postmortem examination results in 351 full‐term infants with NE, early seizures or both to understand the timing of the insult. They differentiated between lesions acquired antenatally, intrapartum and in the early postpartum period. They reported evidence of an acute insult without established injury or atrophy in most infants, with MRI evidence of established injury in ≤3%. Therefore, they concluded that events in the immediate perinatal period were the most important in neonatal brain injury.^6^ However, it should be noted that their selection of cases had to meet at least three of the following five criteria: late decelerations or meconium staining; delayed onset of respiration; umbilical arterial pH <7.1; Apgar score of <7 at 5 minutes; and multi‐organ failure. The inclusion criteria were geared towards finding cases related to acute peripartum problems. This was not a population‐based study, and it is possible that cases without intrapartum factors as detailed above were not studied in the first place.

Korst et al. reported on 47 term infants with NE who experienced prolonged bradycardia that lasted until birth and showed evidence of permanent neurological injury. Only 10 infants exhibited all four features of profound hypoxemia: arterial pH <7.0, 5‐minute Apgar ≤3, seizures within 24 hours of birth and multi‐organ failure. This study demonstrated that even profound hypoxia leading to damage does not necessarily exhibit all these features.^7^ It appears that permanent injury can occur even with lesser degree of hypoxia if the baby is particularly susceptible.

On the other hand, epidemiological studies on cerebral palsy in term neonates have identified that peripartum hypoxia was identified in approximately 15% (range, 8%–28%).^8^ It is known that not all cases with HIE result in cerebral palsy. In summary, it is reasonable to conclude that the proportion of infants with hypoxia/ischemia among those with clinical features of NE is not as high as 90%, but equally not as low as 5%. Further well‐designed prospective studies are needed to clarify accurate incidence of hypoxia/ischemia among neonates who present with NE.

## 2 WHAT IS THE MOST APPROPRIATE TERM TO DESCRIBE A NEONATE WITH CLINICAL FEATURES SUGGESTIVE OF AN ALTERED NEUROLOGICAL STATE?

There is an urgent need to standardize documentation of a neonate who presents with clinical features of an altered neurological state. The terms used included HIE, birth asphyxia, NE, “depressed infant”, etc. It has been argued by some authors that, for several reasons, the most appropriate term to use is NE.^9^, ^10^ It is often not known whether the etiology is a hypoxic/ischemic insult or due to other causes. The blanket use of the term HIE assumes etiology, which is neither correct nor necessary. Such practice may create obstacles in clinical practice and research by assuming that the exact etiology is already known and further delineation of cause of NE is unnecessary. Moreover, this may encourage the practice of defensive medicine by maternal care providers.

It is acknowledged that NE has many other causes apart from hypoxia: intracranial hemorrhage, hypoglycemia, severe hyperbilirubinemia, metabolic disorders, malignant epileptic syndromes, neurodegenerative disorders, and intracranial infection among others.^11^ However, in an opinion paper, Volpe^12^ argued that, for an infant with clinical features of encephalopathy, hypoxia/ischemia as a cause of NE could be suspected in the presence of arterial cord blood gases showing significant acidosis, lower Apgar scores and imaging features consistent with acute hypoxic‐ischemic injury. Timely suspicion is important because “therapeutic hypothermia (TH)” is an identified therapy for such neonates which can limit injury by hypoxia/ischemia and reperfusion.^13^ The window of opportunity for maximal benefit with TH is within 6 hours of the injury. Prompt identification of cases of NE due to hypoxia/ischemia has the advantage of selecting neonates most likely to benefit with this therapy.^14^ At the same time, it is appropriate to use the umbrella term NE for cases of encephalopathy without convincing features of hypoxia/ischemia, and HIE be listed as a potential reason for specific cases of NE. When evidence such as low arterial pH, a sentinel event or metabolic acidosis is present, it would be difficult to argue that timely intervention would not have altered the outcome.

Therefore, we urge that all neonates with clinical features of NE should not be designated as HIE, and clinicians should avoid the use of alternate terminologies. When in doubt, clinicians should describe the state of infant without providing any implicating or insinuating notes and refrain from documenting the reason for NE until complete investigations. They should also be careful in their counseling of the parents. We recommend that, whenever possible, a joint counseling by maternal and neonatal care providers will allow better contextualization for parents as to the condition, progress and plan for their infant. It would be reasonable to outline for parents the clinical findings, the different pathogenetic mechanisms in general that can be responsible for such a clinical state and what further management (investigations and treatment) plans will be in place over next few days to help clarify the picture. It should be acknowledged that event(s) leading to NE may not be peripartum, may not be modifiable and unfortunately, at times unknown. A cautionary note will be that absence of evidence of features suggestive of hypoxemia‐ischemia (low pH or abnormal acid‐base status) does not exclude HIE, since susceptibility/tolerance to even milder degree of hypoxemia may be variable among different fetuses based on preexisting state (eg chronic placental insufficiency). At the same time, NE with features suggestive of hypoxia‐ischemia is most appropriately called hypoxic‐ischemic encephalopathy. It should be promptly recognized, as earlier intervention with TH can help prevent progression of brain injury.
